# Poly(d,l-lactide-*co*-glycolide) (PLGA) Nanoparticles Loaded with Proteolipid Protein (PLP)—Exploring a New Administration Route

**DOI:** 10.3390/polym12123063

**Published:** 2020-12-21

**Authors:** Alexandre Ferreira Lima, Isabel R. Amado, Liliana R. Pires

**Affiliations:** INL–International Nanotechnology Laboratory, Avenida Mestre José Veiga s/n, 4715-330 Braga, Portugal; alexandre98.lima@gmail.com

**Keywords:** dissolving microneedles, multiple sclerosis, PLP, transdermal delivery, PLGA

## Abstract

The administration of specific antigens is being explored as a mean to re-establish immunological tolerance, namely in the context of multiple sclerosis (MS). PLP139-151 is a peptide of the myelin’s most abundant protein, proteolipid protein (PLP), which has been identified as a potent tolerogenic molecule in MS. This work explored the encapsulation of the peptide into poly(lactide-*co*-glycolide) nanoparticles and its subsequent incorporation into polymeric microneedle patches to achieve efficient delivery of the nanoparticles and the peptide into the skin, a highly immune-active organ. Different poly(d,l-lactide-*co*-glycolide) (PLGA) formulations were tested and found to be stable and to sustain a freeze-drying process. The presence of trehalose in the nanoparticle suspension limited the increase in nanoparticle size after freeze-drying. It was shown that rhodamine can be loaded in PLGA nanoparticles and these into poly(vinyl alcohol)–poly(vinyl pyrrolidone) microneedles, yielding fluorescently labelled structures. The incorporation of PLP into the PLGA nanoparticles resulted in nanoparticles in a size range of 200 µm and an encapsulation efficiency above 20%. The release of PLP from the nanoparticles occurred in the first hours after incubation in physiological media. When loading the nanoparticles into microneedle patches, structures were obtained with 550 µm height and 180 µm diameter. The release of PLP was detected in PLP–PLGA.H20 nanoparticles when in physiological media. Overall, the results show that this strategy can be explored to integrate a new antigen-specific therapy in the context of multiple sclerosis, providing minimally invasive administration of PLP-loaded nanoparticles into the skin.

## 1. Introduction

The use of antigen-specific therapies has been explored for the treatment of autoimmune diseases. The premise is to selectively disarm autoimmune responses without suppressing global immunity [1]. Multiple sclerosis (MS) is an autoimmune and demyelinating disease characterized by the presence of inflammatory infiltrates (T cells, B cells, macrophages) within the central nervous system (CNS) that leads to immune-mediated myelin and axonal damage. In the case of MS, peptides from the three major myelin proteins—myelin basic protein (MBP), myelin oligodendrocyte glycoprotein (MOG), and proteolipid protein (PLP)—have been identified to be related to autoimmunity. The use of peptides from myelin proteins to restore immunological tolerance has been extensively investigated, showing positive results in experimental autoimmune encephalomyelitis (EAE) animal models and also in clinical trials.

In the early studies, peptides such as PLP139-151 were chemically crosslinked at the surface of syngeneic splenic leukocytes using ethylene carbodiimide. The infusion of these modified cells induced antigen-specific immune tolerance, overcoming drawbacks in trials directly related to the delivery of soluble peptides or antibodies [2]. However, the direct administration of engineered cells involves limitations and relevant costs for cell isolation under good manufacturing practice (GMP) conditions. In this context, and to translate the technology into the clinical practice, the delivery of antigen crosslinked micro- and nanoparticles was explored. Additionally, the use of nano- and microparticles takes advantage of the intrinsic properties of biomaterials and nanoparticulate systems, which can enhance delivery and cell targeting, namely toward dendritic cells, which play a critical role in the immune response [3].

The intravenous administration of poly(d,l-lactide-*co*-glycolide) (PLGA) microparticles crosslinked with a PLP139-151 peptide (the immunodominant T cell myelin epitope in SJL mice from the myelin’s most abundant protein, PLP) demonstrated an ability not only to reduce the clinical score when administrated prophylactically, but also to treat the disease [4]. Interestingly, the same peptide administered in the form of colloidal hydrogel was effective only if administered before disease onset [5]. The incorporation of poly(ethylene-*co*-maleic acid) (PEMA) as a surfactant in a PLGA formulation allowed the preparation of smaller nanoparticles, providing a reliable platform for different antigen crosslinking, as demonstrated by the relevant results in the induction of immunological tolerance both in the context of EAE [6] and in a transplantation model [7]. Alternatively to antigen crosslinking, nanoparticles can be loaded with an antigen of interest. This concept can be extended to the development of multifunctional systems that combine the delivery of antigens with the encapsulation of other molecules/drugs as a means to make the immune response more specific and/or more effective. Relevant results were obtained by Yeste et al., loading gold nanoparticles with the antigen (MOG35-55) along with a tolerogenic molecule, ITE (2-(1′H-indole-3′-carbonyl)-thiazole-4-carboxylic acid methyl ester). The authors showed that the combination could achieve functional regulatory T cells in an EAE animal model more efficiently than MOG-loaded particles [8]. A different study reported treatment with PLGA nanoparticles containing MOG35-55 and interleukin-10 (IL-10). Although the results in terms of regulatory T cell expansion were not as impressive as those obtained for crosslinked nanoparticles, the nanoparticles caused a reduction in the severity of the disease via subcutaneous administration [9].

Microneedles have been investigated for the minimally invasive delivery of drugs through the skin, overcoming the *stratum corneum* barrier [10,11,12]. Early reports showed improved immunogenicity of molecules when administered via microneedle devices [13]. This promising result was considered to be related to delivery at the epidermal and intradermal layers of the skin, which is highly rich in immunologically active antigen-presenting cells (APCs). These cells deliver antigens to the proximal lymph nodes where T and B cells are activated, triggering an immune response [14]. Additionally, in the skin (particularly in the epidermis and the epithelium from the hair follicles), monocytes and Langerhans cells are abundant Langerhans cells display intrinsic tolerogenic properties in vivo [15].

To take advantage of skin immunogenicity, the use of microneedle patches is presented as an interesting minimally invasive means to deliver molecules that target the immune system [11,13]. In previous work, we designed and prepared dissolvable microneedles for the transdermal administration of molecules [16,17]. We showed the incorporation of the PLP139-151 peptide and its release at therapeutic doses after 3 days in physiological media [16]. As means to improve peptide stability and add a second layer of control to the release of the molecules, in the present work, we explore the encapsulation of the PLP peptide in PLGA nanoparticles and subsequent incorporation into microneedles.

## 2. Materials and Methods

### 2.1. Materials

Poly(d,l-lactide-*co*-glycolide) (PLGA) and acid terminated PLGA (PLGA.H) (50:50) were kindly offered from PURASORB (Corbion, Amsterdam, The Netherlands). Ethyl acetate 99.8%, poly(vinyl alcohol) (PVA, Mowiol 18–88, Mw ~130000), poly(vinyl pyrrolidone) (PVP, Mw ~10000), rhodamine 6G, and trehalose were purchased from Sigma-Aldrich (Saint Louis, MO, USA). The PLP139-151 peptide with the sequence HSLGKWLGHPDKF (purity >95%) was purchased from Primm Biotech (Cambridge, MA, USA) and stored at −80 °C in a 50 mg.ml^−1^ stock solution in water.

### 2.2. PLGA Nanoparticle Preparation

PLGA nanoparticles were prepared by double emulsion based on a previously described procedure [18]. Briefly, PLGA (PLGA20: 20 mg; PLGA60: 60 mg) was dissolved in 2 mL of ethyl acetate and sonicated for 15 s (70% amplitude) using an ultrasonic homogenizer (Branson Digital Sonicator, Saint Louis, MO, USA), resulting in a w/o emulsion. An equal volume of PVA solution (7% (*w/v*) in water) was added and sonicated for an additional 30 s (70% amplitude), resulting in a w/o/w double emulsion. The organic solvent was evaporated in a Savant SPD121P vacuum centrifuge (Thermo Fisher Scientific, Waltham, MA, USA) at 10,000 rpm for approximately 1 h at 40 °C. Fluorescent nanoparticles were prepared, adding rhodamine (0.5 mg·mL^−1^, 1 mg) to the organic solution. To prepare peptide-loaded nanoparticles, 400 µg of PLP139-151 was added to the PLGA organic solution. The prepared nanoparticle suspensions were stored at 4 °C until further use.

### 2.3. Nanoparticle Freeze-Drying, Stability, and Storage

The nanoparticle suspensions were freeze-dried as follows. To remove debris and non-encapsulated materials, the nanoparticle suspensions were diluted and subsequently centrifuged at 15,000 rpm at 4 °C for 1 h. The precipitated nanoparticles were resuspended in the same volume of water or trehalose 10% (*w/v*) solution before freeze-drying for 24 h (LyoQuest, Telstar, Terrassa, Spain).

The nanoparticle suspensions prepared to load in the microneedle patches were frozen without dilution. In brief, 1 mL of nanoparticle suspension was added to 0.5 mL of trehalose 10% (*w/v*) and subsequently freeze-dried.

### 2.4. Characterization of Nanoparticles by Dynamic Light Scattering (DLS)

PLGA nanoparticles were characterized in terms of size and zeta potential by dynamic light scattering (DLS) using an SZ–100 nanoparticle analyzer series from Horiba Scientific. Measurements were performed using diluted nanoparticle suspensions (20×) at 25 °C. Size measurements were performed at 90° and assessed in triplicate. Zeta-potential measurements were also performed in triplicate.

### 2.5. Polymeric Microneedle Preparation

Polydimethylsiloxane (PDMS) molds were obtained from silicon masters, as previously described [16]. The 3 cm × 3 cm masters contained 33 × 33 needles with a 600 μm tip-to-tip distance, a height of approximately 600 μm, and a diameter of 200 μm.

To prepare the polymeric microneedles, mixtures of PVA and PVP were prepared. The PVA (10% (*w/v*) in water) and PVP (15% (*w/v*) in phosphate buffer 0.1 M, pH 7.4) solutions were mixed at a 3:2 (v:v) ratio [16]. The mixture was then poured in the central part of the mold. After applying a vacuum for 20 min and drying about 1 h, more PVA:PVP mix was added, covering all the mold (including the borders to facilitate handling). After eliminating all the air bubbles, the polymeric patches were allowed to dry at room temperature for 24 h. The MN patches were peeled off and observed under optical microscopy (Nikon Eclipse Ni-E, Isaza, Portugal). Polymeric microneedle size was assessed from the optical microscopy images of at least 15 needles from three independent patches.

For the preparation of patches loaded with nanoparticles, freeze-dried nanoparticles were resuspended in 0.7 mL of a PVA:PVP mixture and, after overnight under-rotation at 4 °C, were then added to the PDMS mold, as described above.

### 2.6. Rhodamine Loading and Release

The association efficiency of rhodamine 6G in PLGA nanoparticles was determined by quantifying the free molecule in the supernatant after centrifugation at 15,000 rpm for 1 h at 4 °C (MIKRO 200R, Hettich, Kirchlengern, Germany). The fluorescence was assessed at λex = 570 nm and λem = 595 nm using a microplate reader (Synergy H1MFD box, Biotek, Shoreline, WA, USA). The nanoparticles were incubated for 24 h in a phosphate buffer (0.1 M, pH 7.4) at 37 °C, and the released rhodamine was again determined after a centrifugation step.

### 2.7. PLP Quantification–Loading and Release

To quantify the PLP139-151 peptide, a high-performance liquid chromatography (HPLC) analysis was conducted using a 1260 Infinity II LC System (Agilent Technologies, Madrid, Spain) with diode-array detection (DAD) detection. The separation was performed in a PEPTIDE XB-C18 column (3.6 μm, 150 mm × 21 mm, Aeris, Phenomenex, Alcobendas, Spain). The mobile phase consisted of a mixture of acetonitrile with 0.1% trifluoroacetic acid (TFA; A) and water containing 0.1% TFA (B). Gradient elution was performed at a constant 0.3 mL/min^−1^ flow rate from 100% A to 35% during 20 min, followed by isocratic elution at 75% B for 3 min. A post-run of 5 min was conducted to equilibrate the column at the initial mobile phase composition. PLP was detected at 220 nm after a 50 μL sample injection.

PLP loading in the nanoparticles was calculated after the quantification of the PLP detected in the supernatant of centrifuged nanoparticles (15,000 rpm, 1 h, 4 °C).

To assess the release of PLP from the microneedle patches under physiological conditions, the patches were dialyzed (3 kDa Membrane, Orange Scientific, Braine-l’Alleud, Belgium) against a phosphate buffer (0.1 M, pH 7.4) at 37 °C. The medium was collected and refreshed at different time points (4, 24, and 48 h). The collected samples were filtered (0.45 μm) and stored at −20 °C until further analysis. The amount of PLP in the polymeric patches and PLGA nanoparticles was interpolated from a calibration curve of the purified peptide, ranging from 50 to 0.1 μg·mL^−1^.

### 2.8. Statistical Analysis

Statistical analysis was performed using the software Graphpad Prism version 8.0 (GraphPad, San Diego, CA, USA). Statistical differences were calculated using one-way ANOVA followed by Dunnett’s tests for multiple comparisons. A *p* < 0.05 was considered statistically significant and is denoted by “*”, whereas *p* < 0.001 is denoted by “**”.

## 3. Results and Discussion

### 3.1. PLGA Nanoparticles

In the development of new drug delivery systems for antigen-specific therapies, different strategies using nanoparticles have shown promising results due to the properties of the systems. However, incorporating these strategies into macro drug delivery systems that can assure a painless administration of these nanoparticles and antigens brings a novelty to the real-world drug administration scene that surpasses the novelty of the nano. To achieve this goal, we herein prepared and characterized PLGA nanoparticles, subsequently loaded with the PLP139–151 peptide, to an immunodominant T cell myelin epitope found in multiple sclerosis.

**Figure 1:**
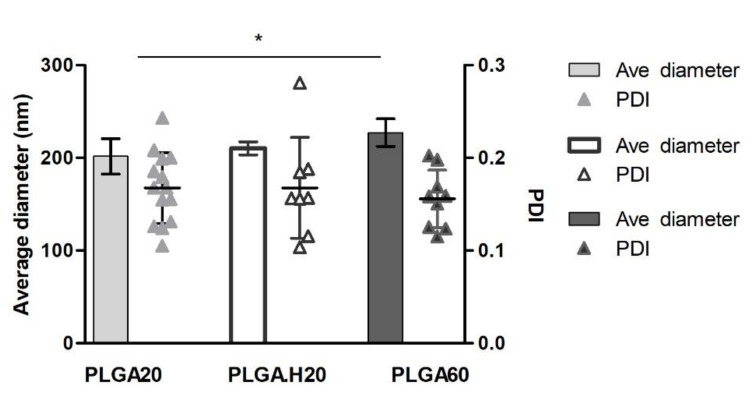
Characterization of poly(lactide-*co*-glycolide) (PLGA) nanoparticles by average diameter and polydispersity index (PDI) (*n* = 3, measured in triplicate).

In the literature, one can find different procedures for the preparation of PLGA nanoparticles and also different outcomes of the synthesis [19]. Particles can be prepared with a large diameter range from 50 nm to 1000 nm, depending on the fabrication process and the publication [19,20]. In this work, we opted for a double emulsion technique, based on previous reports [18,21]. Under the experimental conditions set, the PLGA nanoparticles showed an average diameter of around 200 nm (PLGA20: 201.6 ± 19.0 nm; PLGA.H20: 210.4 ± 7.0 nm; PLGA60: 225.6 ± 14.8 nm) (Figure 1). When increasing the amount of PLGA in the formulation (PLGA60), the particles tended to increase their average size (~220 nm) over what is seen in the literature [18,21]. The average polydispersity index was found to be below 0.17, showing the homogeneity of the particles (Figure 1). The prepared nanoparticles held a negative surface charge in the range of −20 mV for all formulations, as measured by assessing the zeta potential. The results are in accordance with the nature of the used materials, as PLGA holds carboxylic acid terminal groups, which are expected not to be protonated in the media, and a residual charge can also be provided by PVA acetate groups.

**Figure 2:**
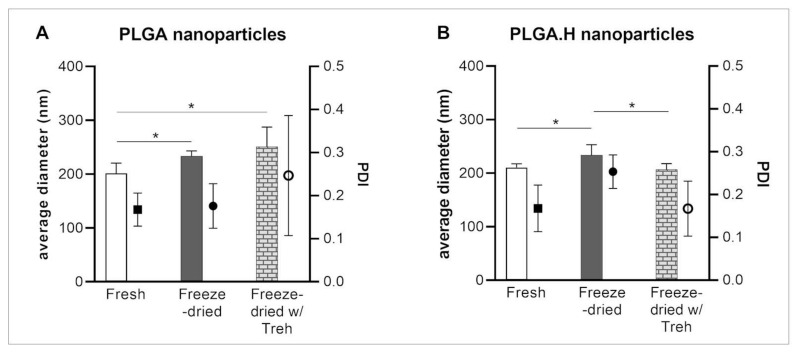
Characterization of the (**A**) PLGA20 and (**B**) PLGA.H20 nanoparticles after freeze-drying with or without trehalose (*n* = 3, assessed in triplicate).

Foreseeing incorporation of the particles into polymeric microneedle patches, it became interesting to study the stability of the particles to a freeze–thaw cycle. Freeze-drying is a technique that allows nanoparticle formulations to be stored in solid form and, consequently, to increase their storage stability [22]. In this process, the presence of cryoprotectants, such as sucrose, trehalose, mannitol, among others, has been explored in diversified types of samples, including polymer nanoparticles, liposomes, and drugs [22,23,24,25]. We tested two formulations: PLGA20 and the acid form of the polymer, PLGA.H20, also exploring the presence of a cryoprotectant, trehalose 10% (*w/v*) [25]. Trehalose was recently shown to be more efficient in preserving protein structure for long-term storage [26]. In general, after freeze-drying, the nanoparticles presented an increased average diameter. This increase was reversed when the process occurred in the presence of trehalose for the PLGA.H nanoparticles (Figure 2A), whereas the same effect was not detected in the PLGA20 formulation (Figure 2B). The particles presented good stability after resuspension, making it possible to consider its inclusion in a polymeric microneedle matrix.

### 3.2. Fluorescent PLGA Nanoparticles into Microneedles

To assess the feasibility of nanoparticle incorporation into polymeric, dissolvable microneedle patches, tests were first performed using fluorescent nanoparticles. Initial attempts were performed with fluorescein, but interestingly, we found that the microneedle composition, namely PVP, reduced fluorescein fluorescence, forming nonfluorescent complexes, as reported in [27]. Alternatively, we explored the incorporation of rhodamine into PLGA nanoparticles.

**Figure 3:**
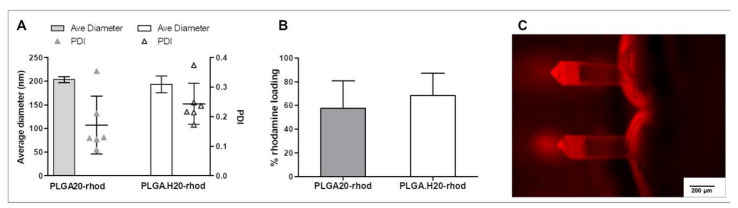
Characterization of PLGA–rhodamine nanoparticles. (**A**) Average diameter of PLGA20 and PLGA.H20 nanoparticles loaded with rhodamine. (**B**) Rhodamine-loading capacity of the PLGA and PLGA.H20 nanoparticles. (**C**) Fluorescence microscopy image of polymer microneedles loaded with PLGA–rhodamine nanoparticles (exposure = 10 ms).

The characterization of the nanoparticles in terms of size and PDI yielded similar results to the empty nanoparticles. Average nanoparticle diameter was around 200 µm (Figure 3A). It was observed that there is a tendency to increase the polydispersity index when loading rhodamine in the nanoparticles; this difference was found to be nonstatistical, however.

When analyzing the loading capacity of the nanoparticles, it was found to be 57.8 ± 23.1% in the PLGA20 formulation and slightly higher, 68.4 ± 18.8%, in the case of PLGA.H20 nanoparticles (Figure 3B). Others report rhodamine loading capacity in PLGA nanoparticles to be around 40–50% [28,29].

The rhodamine-loaded nanoparticles were incorporated into microneedle patches. To do so, the nanoparticles were first freeze-dried and subsequently resuspended into the polymeric matrix. This solution was then applied into the microneedle molds and, after drying, peeled off. As the nanoparticles were fluorescently labeled, it was possible to localize them inside the needle patch structure. Interestingly, the results suggest that nanoparticles might be preferentially located closer to the needle tip, as there was more fluorescence found in that part of the structure (Figure 3C). Particles cannot be distinguished in the structure, and, in fact, it cannot be excluded that free rhodamine was present in the needles. Fluorescence signal in other fluorescence channels can only be obtained with higher exposures, pointing out the specificity of rhodamine detection.

### 3.3. Microneedle Loaded with PLP–PLGA Nanoparticles

**Figure 4:**
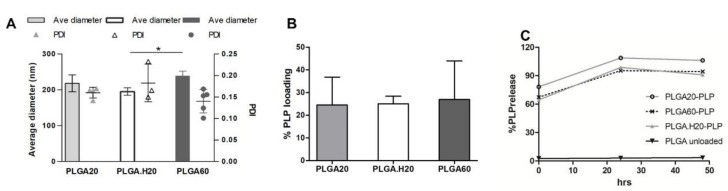
Characterization of PLGA nanoparticles loaded with proteolipid protein (PLP). (**A**) Average diameter and polydispersity index (PDI) of nanoparticles loaded with PLP. (*n* = 3, in triplicate) (**B**) loading efficiency of the different nanoparticle formulations. (*n* = 3) (**C**) Release of the PLP peptide into physiological media, as determined by HPLC (representative experiment out of 2).

The analysis of the physical properties of the PLP–PLGA nanoparticles showed that loading PLP does not significantly change the size of the nanoparticles in comparison with the empty nanoparticle formulation (Figure 4A). A slight increase of about 20 µm in the average size could be pinpointed, but the increase was not statistically significant. Results in Figure 4A also denote that PLGA.H20 (195.1 ± 10.1 nm) showed a significantly smaller size as compared with PLGA60 (238.7 ± 13.6 nm). This same tendency was detected in unloaded PLGA nanoparticles, as discussed above.

The loading of the peptide was found to be on average above 20% for all the formulations tested (Figure 4B). Interestingly, rhodamine loading was significantly higher than for PLP, probably due to the larger size and hydrophobicity of rhodamine that may improve interactions with PLGA. The percentage of peptide PLP encapsulated on PLGA nano- and microparticles was not disclosed in some of the reference publications [6,30]. A higher association efficiency in PLGA particles was described for larger peptides, such as glucagon-like peptide-1 (GLP-1) (67%) [21] or LL37, in which 70% efficiency was achieved in particles above 300 nm [31].

Figure 4C shows the release of PLP from the nanoparticles. The release is very fast, occurring in the first hours/minutes of incubation at 37 °C. Figure 4C represents the initial point at which over 60% of the peptide was not incorporated in the nanoparticles, and, from this point on, the complete release was detected. Release in the first hours of incubation at physiological conditions is also reported in the literature [21,32].

With the final goal to develop a system that allows minimally invasive administration, we incorporated the PLP-loaded nanoparticles into polymeric microneedles. Other systems are reported in the literature, such as exploring the use of microneedles coated with nanoparticles [29,32,33] or incorporating them in a dissolvable structure for the delivery of DNA [34].

**Figure 5:**
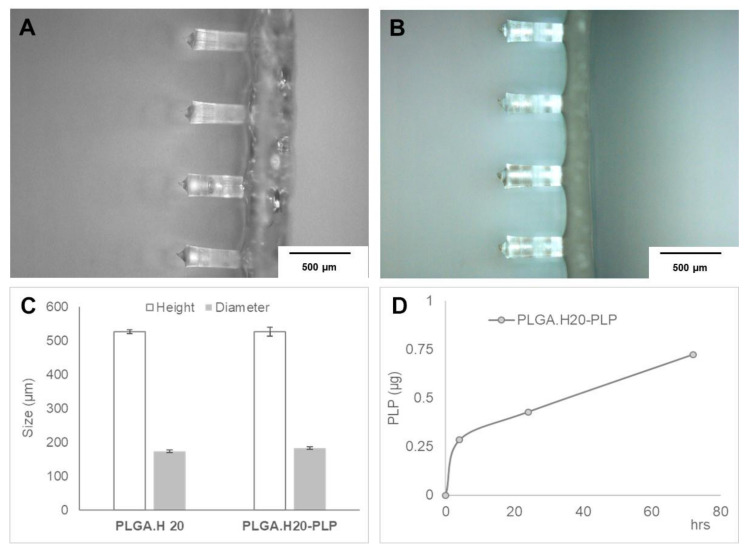
Polymer microneedles loaded with PLGA.H20 nanoparticles. (**A**,**B**) Optical microscopy images of microneedles loaded with (**A**) plain or (**B**) PLP-loaded PLGA.H20 nanoparticles. (**C**) Characterization of the prepared polymer microneedles in terms of diameter and height. (**D**) Quantification of the PLP released from the polymeric system when immersed in PBS a 37 °C, as quantified by HPLC.

The images (Figure 5A,B) show that the incorporation of nanoparticles does not affect the morphology of the microneedles. The prepared patches contained microneedles similar to the ones reported in our previous work [16], being an average height of below 550 µm and a diameter of around 180 µm (Figure 5C). Interestingly, when placed in physiological media, the release of the peptide was likely to occur in a more sustained way (Figure 5D) when compared with the release from the nanoparticles. A burst release was observed in the first period of incubation due to the peptide not being encapsulated or being loosely bound at the surface. The detection of the peptide was challenging due to the presence of large amounts of polymer from the microneedles’ dissolution. To assess the release of the peptide, the patches were maintained in a dialysis bag in physiological media. This procedure aimed to reduce the amount of polymer from the patch that was also released to the media. However, smaller polymer chains can cross the dialysis membrane making it more difficult to detect small amounts of peptide. Thus, in these experiments, the PLP signal in the HPLC chromatogram was possible to isolate only for the microneedle patches containing PLGA.H20 nanoparticles. The amount of PLP quantified was below 1 µg, representing about 2% of the theoretical amount of PLP expected in the sample. If on one side, the graph (Figure 5D) suggests that the release is in its increasing phase, further experiments using higher amounts of loaded nanoparticles and, consequently, of the peptide could help to isolate and quantify the PLP within the polymer mixture. Still, the presented results show that the incorporation of PLGA nanoparticles loaded with PLP onto dissolvable microneedles could bring a new approach to the delivery of antigen-specific therapies.

## 4. Conclusions

This study presented the development of a dissolvable microneedle patch in which PLGA nanoparticles containing PLP were loaded. The nanoparticles showed good stability and the PLP peptide was successfully incorporated.

Although the dose of PLP detected in the microneedle patches was still beyond the therapeutic doses described in the literature, this strategy brings novelty to the administration of antigen-specific therapies, namely in the context of multiple sclerosis.
